# Spatiotemporal brain dynamics of auditory temporal assimilation

**DOI:** 10.1038/s41598-017-11631-0

**Published:** 2017-09-12

**Authors:** Naruhito Hironaga, Takako Mitsudo, Mariko Hayamizu, Yoshitaka Nakajima, Hiroshige Takeichi, Shozo Tobimatsu

**Affiliations:** 1 Department of Clinical Neurophysiology, Neurological Institute Faculty of Medicine, Graduate School of Medical Sciences, Kyushu University, 3-1-1 Maidashi, Higashi-ku, Fukuoka, 812-8582 Japan; 2 Department of Anesthesiology and Critical Care Medicine, Graduate School of Medical Sciences, Kyushu University, 3-1-1 Maidashi, Higashi-ku, Fukuoka, 812-8582 Japan; 3 Department of Human Science/Research Center for Applied Perceptual Science, Faculty of Design, Kyushu University, 4-9-1 Shiobaru, Minami-ku, Fukuoka, 815-8540 Japan; 4 Computational Engineering Applications Unit, Advanced Center for Computing and Communication (ACCC), RIKEN, 2-1 Hirosawa, Wako, Saitama, 351-0198 Japan

## Abstract

Time is a fundamental dimension, but millisecond-level judgments sometimes lead to perceptual illusions. We previously introduced a “time-shrinking illusion” using a psychological paradigm that induces auditory temporal assimilation (ATA). In ATA, the duration of two successive intervals (T_1_ and T_2_), marked by three auditory stimuli, can be perceived as equal when they are not. Here, we investigate the spatiotemporal profile of human temporal judgments using magnetoencephalography (MEG). Behavioural results showed typical ATA: participants judged T_1_ and T_2_ as equal when T_2_ − T_1_ ≤ +80 ms. MEG source-localisation analysis demonstrated that regional activity differences between judgment and no-judgment conditions emerged in the temporoparietal junction (TPJ) during T_2_. This observation in the TPJ may indicate its involvement in the encoding process when T_1_ ≠ T_2_. Activation in the inferior frontal gyrus (IFG) was enhanced irrespective of the stimulus patterns when participants engaged in temporal judgment. Furthermore, just after the final marker, activity in the IFG was enhanced specifically for the time-shrinking pattern. This indicates that activity in the IFG is also related to the illusory perception of time-interval equality. Based on these observations, we propose neural signatures for judgments of temporal equality in the human brain.

## Introduction

Keeping track of time is an essential cognitive ability governed by synchronisation of neural networks in the brain. Different neural mechanisms are involved in supra- and sub-second temporal processing^1–3^, and related systems are involved in complex cortical attentional/cognitive activation^4^. In the literature, motor timing and cognitively controlled timing show clear distinctions^4–6^. Motor timing refers to the timing of output behaviour, such as the temporal organisation of motor acts, and is thought to be generated by external pacing tasks. In contrast, cognitively controlled timing refers to the perceptive aspects of time management generated by internal pacing tasks, such as perceiving and estimating temporal intervals.

To explore the cortical network for cognitively controlled temporal judgment in the human brain, we developed a paradigm that induces “auditory temporal assimilation (ATA)”. Under ATA, the duration of two successive intervals (T_1_ and T_2_), marked by three auditory stimuli, are biased to be perceived as equal even when they are different to a degree that seems enough to be detected (Supplementary Audio 1–3 and Fig. S1). Individuals often perceive the two intervals as equal within a range of −100 ≤ T_1_ − T_2_ ≤ 50 ms, provided that T_1_ ≤ 200 ms. The magnitude of the ATA is markedly greater when the first interval is shorter than the second one (but not vice versa). This is the result of the “time-shrinking illusion,” in which the subjective duration of T_2_ “shrinks” and becomes close to T_1_^7, 8^. This asymmetric assimilation cannot be attributed to the uncertainty of discrimination, and should be attributed to a kind of categorical perception^9^. The ATA paradigm requires perceiving and estimating the equality of two temporal intervals without using motor control, suggesting that a “cognitively controlled time measurement system” is responsible^5^. Thus, ATA can provide an insight into human sub-second cognitive temporal processing. Our previous electrophysiological studies^10, 11^ demonstrated that event related potentials (ERPs) in the frontal regions were related to the ATA; the contingent negative variation appeared within 100 ms during T_2_ and the slow negative component related to temporal judgment appeared within 80 ms after T_2_. Based on these results, we hypothesised that neuronal activity in the localized brain areas during and after stimulus presentation would be related to the brain mechanism that underlies ATA.

Converging evidence from recent neuroimaging studies points to several regions in the frontal cortex as key structures related to temporal tasks. The right dorsolateral prefrontal cortex appears to be involved in the comparison of time intervals^12^, while the inferior frontal gyrus (IFG) is implicated in judging durations^13^. Additionally, the right inferior parietal lobe (IPL) is activated during temporal processing^14, 15^. The temporoparietal junction (TPJ), a core anatomical locus within the IPL, is considered the common substrate for sub- and supra-second perceptual timing tasks^16^. The frontoparietal cortices have been reported to be involved in cognitively controlled timing, especially those in the right hemisphere (e.g., the right dorsolateral prefrontal cortex and the right parietal cortex). Also, activation of the supplementary motor area (SMA) and the right IFG have consistently been found in time-perception studies^17^.

Our goal was to clarify the neural mechanism underlying the time-shrinking illusion under ATA and to expand our understanding of human sub-second cognitive temporal processing. To determine the precise spatiotemporal dynamics of ATA, we focused on the frontal and parietal areas that are assumed to be related to encoding and judgment in cognitive time estimation. This was because our previous psychophysical and electrophysiological studies of ATA^7, 10, 11, 18^ suggested that encoding and judgment processes via the frontal and parietal areas are the key signatures of ATA.

## Results

### Stimuli and behavioural results

**Figure 1:**
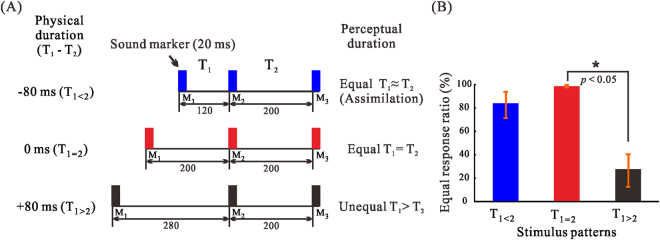
(**A**) A schematic illustration of the auditory stimulus patterns (M; marker, T; time). (**B**) Behavioural results of equality judgments. Equal response ratios of the three different stimulus patterns. T_1_ was perceived as nearly equal to T_2_ when T_1<2_ (T_1_ − T_2_ = −80 ms), but not when T_1>2_ (T_1_ − T_2_ =  +80 ms).

We adopted a task using two consecutive, short-duration (millisecond) intervals (T_1_ and T_2_) that were defined by three standard auditory markers (tone bursts). Figure 1 shows schematic images of the stimulation and the behavioural results. We included three stimulus patterns as follows: T_1_ < T_2_ (T_1_ = 120 ms, T_2_ = 200 ms; T_1<2_), T_1_ = T_2_ (200 ms, 200 ms; T_1=2_), and T_1_ > T_2_ (280 ms, 200 ms; T_1>2_) (Fig. 1A and Supplementary Audio 4–6). Hereafter, we use the three symbols T_1<2_, T_1=2_, and T_1>2_ to denote the three different stimulus patterns. A previous behavioural study^7^ found that a T_1<2_ reliably induced ATA, while a T_1>2_—having the same absolute difference in duration—did not. In judgment conditions, the participant indicated whether or not the T_1_ and T_2_ durations were equal by pressing one of two buttons. In no-judgment (control) conditions, participants listened to the tones and pressed one of the two buttons without making any judgments. Seventeen healthy volunteers (12 males, mean ± SD age; 24.8 ± 4.7 years) participated in the experiment. All participants were right-handed, with no hearing deficits. Informed consent was obtained from each participant after the purpose and procedure of the experiment were explained. The study was approved by the institutional ethics committee of Kyushu University, and was carried out in accordance with the latest version of the Declaration of Helsinki.

Figure 1B shows the results of the behavioural task performed during magnetoencephalography (MEG) in the judgment condition. There was a main effect of stimulus pattern (*F*_2,48_ = 59.03, *p* < 0.001, *η* ^2^ *p* = 0.71). T_1=2_ was rated as equal in 97.3% of cases, but this ratio was smaller for T_1>2_ (*t* = 10.19, *p = *0.001, Cohen’s *d* = 3.71), and not different for T_1<2_ (*t* = 2.11, *p* = 0.06, Cohen’s *d* = 0.95). T_1_ was perceived to be equal to T_2_ when it was shorter than T_2_, but not when it was longer.

### Right hemispheric predominance

**Figure 2:**
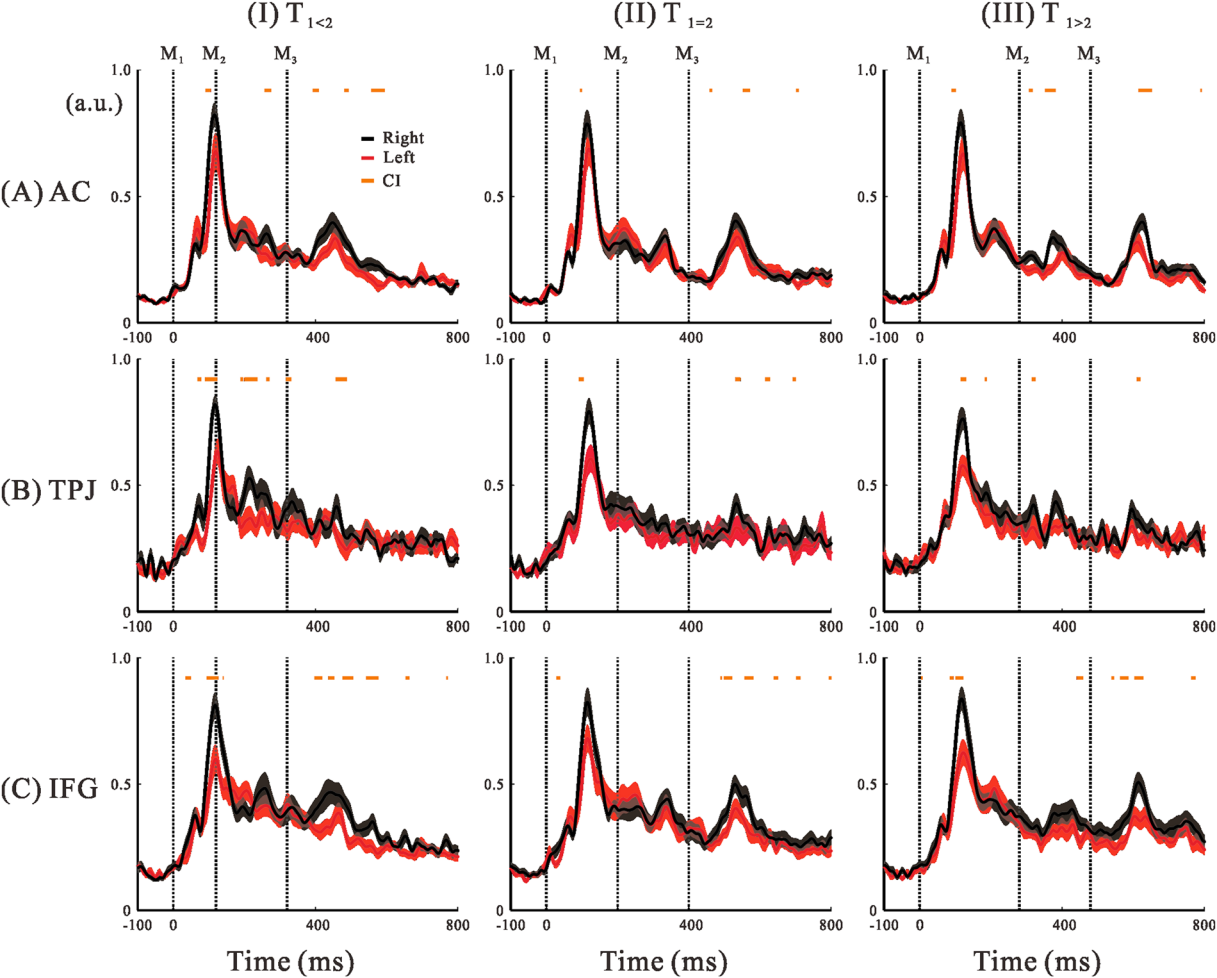
Temporal profiles of the hemispheric differences in regional activities in (**A**) auditory cortex (AC), (**B**) the temporoparietal junction (TPJ), and (**C**) inferior frontal gyrus (IFG) for the three different stimulus patterns (T_1<2_ (I), T_1__=__2_ (II), and T_1>2_ (III)). The timings of the sound markers are plotted as vertical dotted lines and labelled M_1_ (M; marker), M_2_, and M_3_. Black lines represent the time-series data for the normalised group-averaged (n = 17) regional activity for the right hemisphere, and red lines denote those for the left hemisphere. Colour-matched transparent areas represent the Standard Errors (SEs) of the averaged activations. Orange horizontal bars presented the time intervals at which the confidence intervals (*CI*s) of laterality index (*LI)* were greater than zero.

First, we assessed whether or not hemispheric predominance was present. The five-way repeated measures analysis of variance (ANOVA) (see Supplementary Tables S2 and S3 for more detail) revealed that the main effect of hemisphere (*F*_1,16_ = 10.02, *p* < 0.007, *η* ^2^ *p* = 0.39) was significant. Because right hemispheric activation over the individual time points indicated right hemispheric predominance for the three inspection Regions of Interest (iROIs), we further assessed the time-dependent effects of hemisphere and experimental condition based on the confidence interval (*CI*) of the laterality index (*LI;* equation 3). Figure 2 shows the group-averaged regional activity in three iROIs for three stimulus patterns in the left (red) and right (black) hemispheres. At several time points the lower limits of the *CI*s were greater than zero (orange horizontal bars). Hereafter, we use the three symbols M_1_, M_2_, and M_3_ to denote the first, second, and third marker, respectively. The *CI*s of *LI* also indicated right hemispheric predominance in the auditory cortex (AC) in response to M_1_ and M_3_ (Fig. 2A). In the TPJ, right predominance of responses to M_1_ was observed but no obvious peak activations were seen after M_3_. Instead, dominant activation in the right hemisphere was found for T_1<2_ and T_1>2_ patterns during T_2_ (Fig. 2B). In the IFG, *CI*s of *LI* for responses to M_1_ and M_3_ indicated right hemispheric predominance for all three-stimulus patterns, and was evident when compared with the AC and TPJ (Fig. 2C). The consistent right hemispheric predominance of responses in the IFG after M_3_ contrasted with the TPJ responses that showed clear right-predominance only during T_2_. One important observation here is that right predominance occurred within specific time periods, as suggested by the *CI* representations, but not by simple baseline shifting.

### Judgment vs. no-judgment conditions

**Figure 3:**
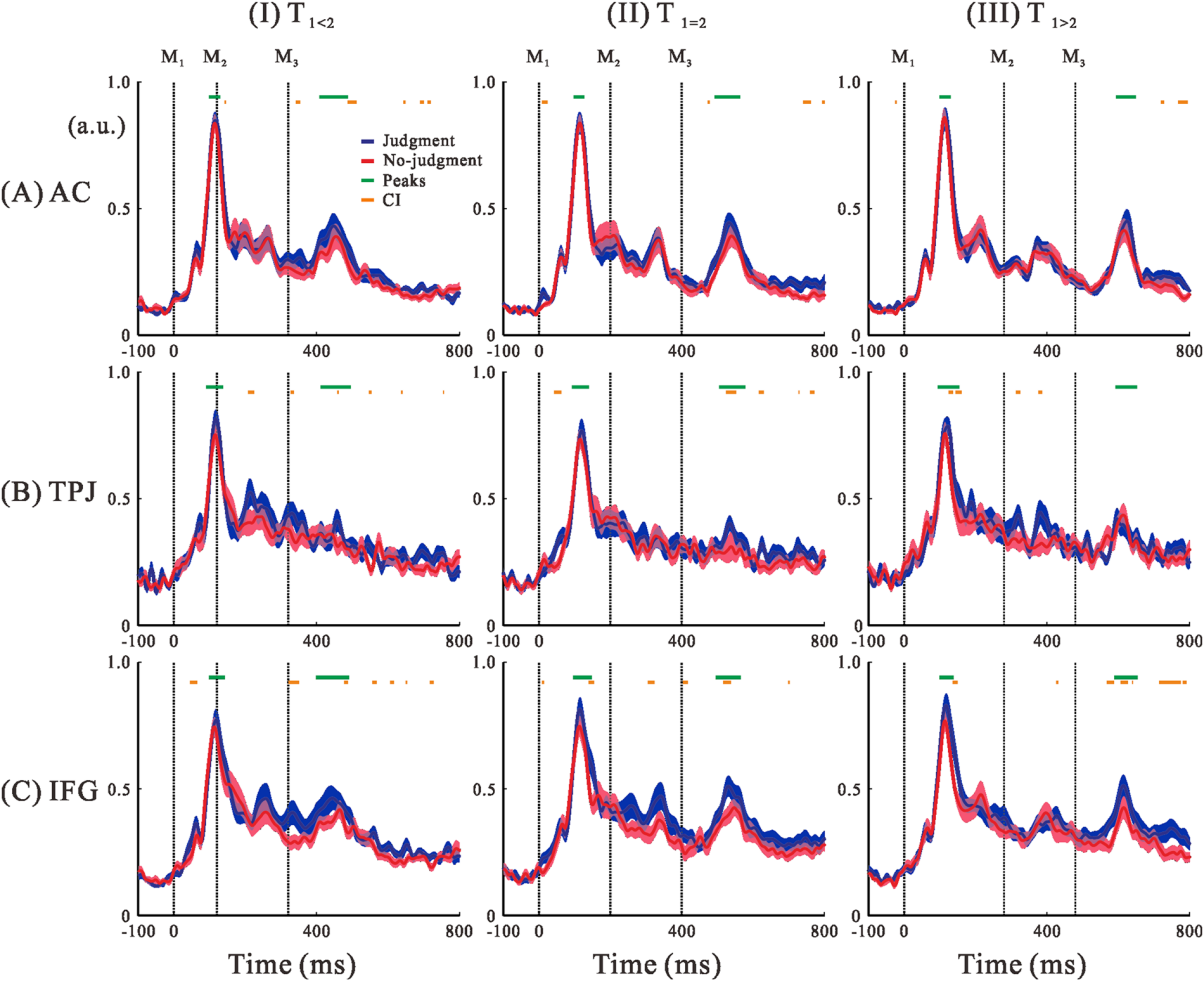
Temporal profiles of right hemispheric activation in the AC (**A**), TPJ (**B**), and IFG (**C**) for the three different stimulus patterns (T_1<2_ (I), T_1__=__2_ (II), and T_1>2_ (III)). Blue lines represent normalised time-series data for the group-averaged (n = 17) regional activity in the judgment condition, and magenta lines denote those for the no-judgment condition. Colour-matched transparent areas represent the SEs of the averaged activations. Orange horizontal bars presented the time intervals at which *CI*s of judgment index (*JI)* were greater than zero. Green horizontal bars indicate the periods of the selected peak latencies (M_1_ and M_2_ for TPJ, M_1_ and M_3_ for IFG) from each individual for which enlarged activation maps are provided in Fig. 4.

We examined the effect of temporal judgment with an ANOVA, which revealed a significant main effect of condition (*F*_1,16_ = 9.53, *p* < 0.008, *η* ^2^ *p* = 0.37). Given the right hemispheric predominance observed in the crucial time periods (Fig. 2) and the main effect of judgment condition, we assessed how judging the interval durations affected the right AC, IFG, and TPJ based on *CI*s of judgment index (*JI*; equation 4). Additionally, the iROI × stimulus × time interaction was significant (*F*_16, 256_ = 6.67, *p* < 0.0001, *η* ^2^ *p* = 0.29). Figure 3 compares the group-averaged regional activities during judgment and no-judgment conditions. In the AC, no outstanding differences were observed between the two conditions for any of the three stimulus patterns (Fig. 3A). In the TPJ, differences between the two conditions were observed after M_2_ when the two intervals had different durations (Fig. 3B). Enhanced activation during judgments was observed for the T_1<2_ and T_1>2_ conditions during T_2_ (Fig. 3B–I,III). Thus, we focused on the role of the TPJ during T_2_. In the IFG, activation during the judgment condition was higher than during the no-judgment condition, especially after M_3_ (Fig. 3C) in which significant *CI* ranges were observed. Therefore, we analysed the activity after M_3_.

**Figure 4:**
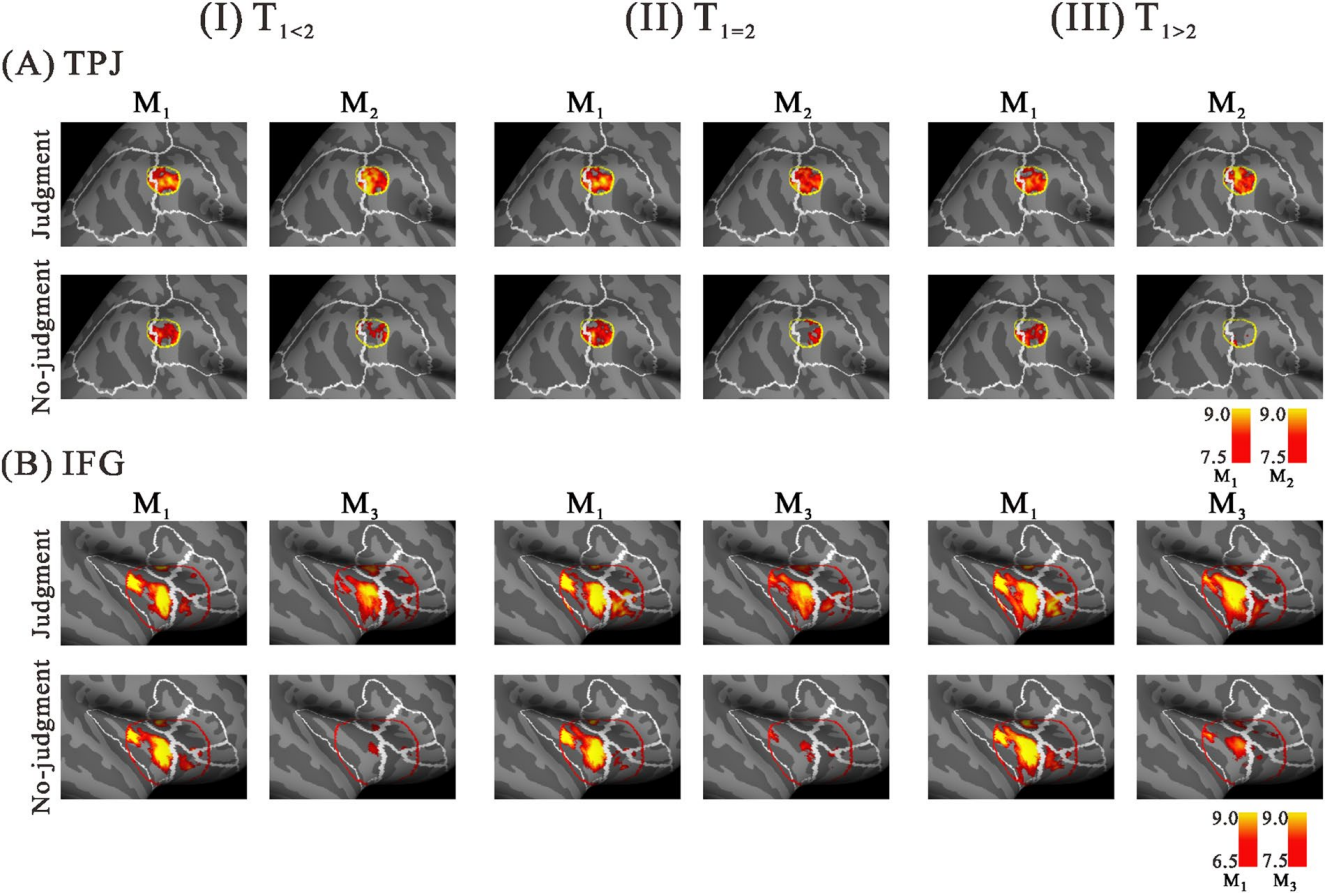
Comparisons of the right hemispheric activation maps between judgment and no-judgment conditions in the TPJ (**A**) and IFG (**B**) for the three different stimulus patterns (T_1<2_ (I), T_1__=__2_ (II), and T_1>2_ (III)). Peak activations of M_1_ and M_2_ for the TPJ and M_1_ and M_3_ for the IFG were averaged over the 17 participants. Each activity corresponded to the peak time ranges represented as green horizontal bars in Fig. 3. The two bars on the right side indicate the colouring threshold levels of the activation maps.

**Figure 5:**
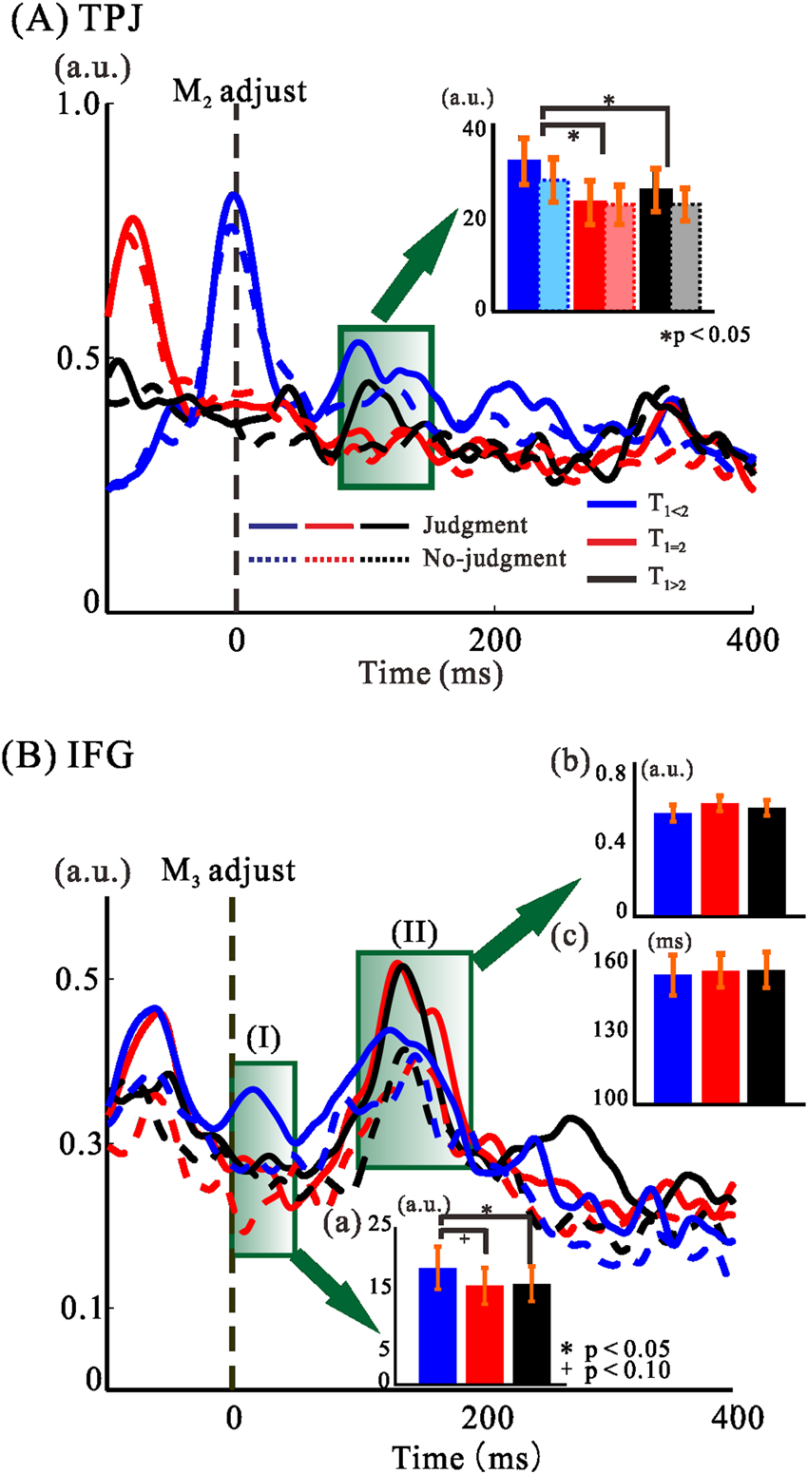
(**A**) Regional activities aligned at M_2_ onset. Solid lines indicate activity during the judgment condition while dotted lines represent activity during the no-judgment condition. The inset bar graph shows the integrated mean amplitudes and SDs of regional activity within the area surrounded by the green square (80–150 ms). Bar values are 33.8 (±5.7) for T_1<2_, 23.8 (±5.4) for T_1__=__2_, and 27.7 (±5.7) for T_1>2_ in the judgment condition, and 28.9 (±6.1), 23.0 (±5.0), and 22.9 (±3.9), respectively, in the no-judgement condition (Table S4). (**B**) Regional activities aligned at M_3_ onset. Time-ranges of peak latencies from 0 to 50 ms (I) and from 101 to 191 ms (II) after M_3_ onset are encapsulated by the green square. Inserted bar graphs represent the integrated amplitude within a 50-ms time window starting at M_3_ onset (a) (Table S4) and peak amplitude (b) and latency (c) that correspond to the response to M_3_ (Table S5). Means (±SDs) in the bar graph (a) are 18.7 (±3.4) for T_1<2_, 15.8 (±2.9) for T_1__=__2_, and 16.1 (±2.8) for T_1>2_. Statistical significance determined by ANOVA are represented within the bar graphs and Table S4.

Figure 4 shows the spatial comparison between the judgment and no-judgment conditions in the TPJ (Fig. 4A) and IFG (Fig. 4B). In the TPJ, task-dependent differences were seen after M_2_, but not after M_1_. Although we did not find clear differences after M_1_, we did observe higher activation patterns in space after M_2_ for T_1_ ≠ T_2_, especially in T_1>2_ (Fig. 4A–I and III). In the IFG, while no condition-dependent enhancement was seen after M_1_, a large difference was observed after M_3_ (Fig. 4B). The estimated activation centres (MNI coordinates) in the IFG iROI were almost identical for the three stimulus patterns (T_1<2_: [32.65, 18.61, −0.4]; T_1=2_: [32.98, 18.22, −0.85]; T_1>2_: [33.46, 17.50, −1.12]), and all were estimated as “G_insular_short” by anatomical annotation. The target latencies for the TPJ and IFG were clarified, and statistical analyses were applied after the time adjustment. Figure 5 plots the summary of these statistical results and we describe them below.

### TPJ responses during the second interval

In Fig. 3, we can see that aside from peak responses to M_1_, TPJ activity differed from AC and IFG activity. Figure 5A superimposes regional activity aligned to the end of M_2_ in the right TPJ. Time ranges for the peak latencies in the judgment condition were from 202 to 262 ms (−120 ms) (blue for T_1<2_), from 258 to 341 ms (−200 ms) (red for T_1=2_), and from 333 to 426 ms (−280 ms) (black for T_1>2_). Note that the negative values inside the brackets are the time-adjustment parameters. The area encapsulated by the green square indicates clear differences between judgment and no-judgment conditions, in which enhanced activation was evident in judgment conditions of physically unequal stimulus patterns. The result of two-way ANOVA on the integrated amplitude 80–150 ms after M_2_ for the judgment and no-judgment conditions (Fig. 5A, green square) showed a significant main effect of stimulus pattern (F_2,32_ = 8.30, *p* < 0.01, *η* ^2^ *p* = 0.342) (Table S4). The integrated amplitude within the 70-ms time range after M_2_ was significantly larger for the T_1<2_ pattern compared with the T_1=2_ (*p* < 0.01) and T_1>2_ (*p* = 0.035) stimulus patterns (Fig. 5A, bar graph). Moreover, activity in the judgment condition was significantly higher compared with the no-judgment condition for the T_1<2_ (*p* = 0.04) and T_1>2_ (*p* = 0.05) patterns. Thus, TPJ activity elicited by unequal stimulus patterns was enhanced during T_2_.

### IFG responses after the third marker

Clear differences in the IFG were noted after M_3_ in all three stimulus patterns, suggesting that IFG activity after M_3_ was related to making temporal judgments. Figure 5B shows superimposed regional activity for each stimulus pattern with respect to the temporal judgment, and aligned at the end of M_3_. For the right IFG, a clear but small peak emerged immediately after M_3_ in T_1<2_ (Fig. 5B–I). ANOVA performed on the integrated amplitude during a 50-ms time window beginning at M_3_ onset revealed a significant main effect of stimulus pattern (F_2,32_ = 4.29, *p* < 0.03, *η* ^2^ *p = *0.21) (Table S4). The integrated regional activity amplitudes for the T_1<2_ pattern were significantly larger than those for the T_1=2_ (*p* = 0.053) and T_1>2_ (*p* = 0.029) patterns (Fig. 5B–a). Peaks were also seen in the second time window, corresponding to the onset of M_3_ in individual stimulus patterns (Fig. 5B–II). Within this time range (II), a one-way repeated-measures ANOVA was performed on the peak amplitude and the latency corresponding to the responses that occurred after M_3_. No significant differences were found in regional activity amplitudes or latencies in the three judgment conditions (Fig. 5 B–b and 5B–c) (Table S5). IFG observations indicated two important points: First, activity after M_3_ was greater in the judgment condition than in the no-judgment condition. Peak latencies and signal power levels were not significantly different across the three stimulus patterns in the temporal profile. Second, the judgment-specific enhanced activity after M_3_ depended on the stimulus pattern; the T_1<2_ pattern elicited higher activity after M_3_ than did the other patterns.

## Discussion

We measured neuromagnetic responses under ATA to determine the neural network related to sub-second cognitive temporal judgments. We found brain activity related to the duration encoding and judgment processes on time perception (*what*), during and after stimulus presentation (*when*), in the right-TPJ and right-IFG (*where and how*). Our interpretation of the results is as follows: The AC receives input and communicates it to the TPJ with right hemispheric predominance. The TPJ receives the tone cue from the AC then encodes the duration and compares the T_1_ and T_2_ durations. The IFG makes a judgment between the two durations. Immediately after the final marker, the IFG engages the critical process related to the time-shrinking illusion, as assumed in the processing time hypothesis^7^. Based on the neuromagnetic signatures of ATA, we discuss our novel findings and propose a hypothesized neural model of “Temporal Judgment Processing for ATA”.

We observed right hemispheric predominance associated with specific time points during temporal processing. This tendency clearly began at the start of auditory processing in the AC, as reflected by the M100 responses to M_1_ (Fig. 2A). Hemispheric specialisation in the AC, which plays a key role in processing tonal pitch perception, is well established^19–21^. Therefore, the right hemispheric predominance in the AC that we observed here could have been caused by the rhythmical characteristics of the sound stimuli^22–24^. Right predominance was also seen in the TPJ during T_2_ when T_1_ ≠ T_2_, and in the IFG after M_1_ and M_3_ (Fig. 2C). Studies have shown that right hemispheric predominance is related to cognitive time estimation^4, 6^. Additionally, the right parietofrontal cortex is activated during duration-discrimination tasks^25, 26^.

With respect to the judgement condition, enhanced activity was observed in the TPJ during T_2_, especially when T_1_ ≠ T_2_ (Fig. 5A). Activity between 80–150 ms after M_2_ may be related to a response to the fixed T_2_ of 200 ms, such as the comparison^27^ of T_1_ in memory and T_2_ under encoding, ramping activity^28^, or post-comparison evoked potential^29^ in the TPJ after M_2_. Although dummy presentations were included within the trial blocks, frequent presentation of the 200-ms interval formed a kind of standard interval that made participants implicitly sensitive to deviations from it. Recently, there have been debates about the relationship between perceived time and neural activities evoked during and after the time interval^27, 29^. There is currently no consensus on which signal represents perceived duration.

The centres of activation in the IFG for the three patterns in the judgment condition were almost identically estimated and occurred around 140 ms after M_3_. The location shifted more to the insular side in all cases, regardless of the stimulus patterns (see Fig. 4B). Right IFG activation is assumed to be an index of the judgment process for duration discrimination, as evidenced by right IFG involvement in judging durations^13^, attentional control^30^, categorical decision^26, 31^, and temporal processing. Additionally, the IFG has been reported to be associated with insular activity in a diversity of timing tasks (within a few seconds or less)^13, 32, 33^. The insular activation seen within our IFG-iROI suggests that this region may also be related to non-specific discrimination in temporal processing^32^. We expand our interpretation and possible speculation for the IFG activation below.

**Figure 6:**
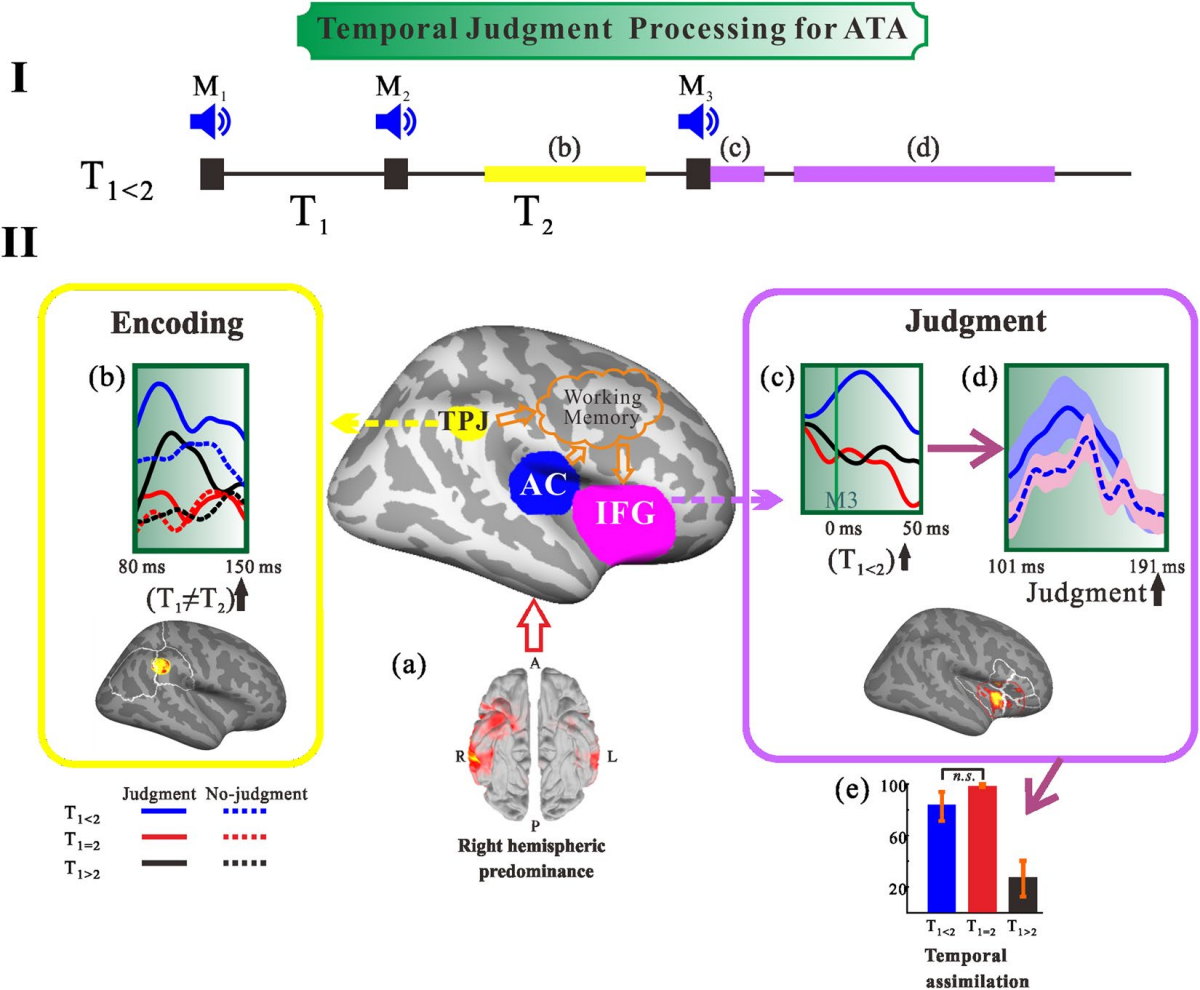
A graphic summary of our findings and a hypothetical illustration of the temporal judgment network for ATA. (**I**) The time course of T_1<2_, which induces the time-shrinking illusion. (**II**) The brain network depicted in this study. (a) to (e) illustrate the sequential processing of ATA. (a) Right hemispheric predominance under ATA. (b) The effect of encoding emerged in the TPJ 80–150 ms after M_2_. (c) Illusory perception just after M_3_. (d) The judgment process in the IFG 140 ms after M_3_. (e) The behavioural results of ATA. Yellow box, TPJ activation; purple box, IFG activation; solid lines, regional activity during the judgment condition; dotted lines, regional activity during the no-judgment condition. T_1<2_ in blue, T_1__=__2_ in red, and T_1>2_ in black.

Figure 6 outlines our findings (encoding process in the TPJ (yellow box) and the judgment process in the IFG (purple box)) and illustrates the hypothetical model for ATA in the case of T_1<2_, making comparisons between stimulus patterns and conditions. Figure 6I shows the T_1<2_ time series and the time ranges b–d indicate the crucial time periods found in this study. We observed right hemispheric predominance for ATA (Fig. 6II–a), as stated in the previous subsection. Comparison of regional activity in the judgment and no-judgment conditions after M_2_ (Fig. 6II, yellow box) showed that TPJ activity was enhanced during T_2_, especially when T_1_ ≠ T_2_. Judgment of the equality of the two consecutive time intervals (Fig. 6II–d), after the critical stage for the illusion (Fig. 6II–c), may follow the encoding or comparison stages in the TPJ (Fig. 6II–b).

Here, we address our speculative insight with respect to one of the underlying mechanisms of ATA. Activation in the right IFG within 50 ms after M_3_ was enhanced when T_1<2_ (Fig. 6II–c). This neuromagnetic observation may underlie our proposed psychophysical model of ATA, or the processing time hypothesis. The main postulates of the processing time hypothesis are that the perceived time is the sum of the physically lapsed duration and the time required for processing the interval in the brain, and that the processing of the interval is constant but is shortened when “quasi-” equality of the intervals is recognised. As the time for processing the interval is estimated to be 80 ms, the hypothesis advocates that the essential part of the assimilation takes place within 80 ms after the third marker^7, 18^. In the T_1<2_ pattern, T_2_ processing is faster than the regular time of 80 ms (Fig. 6I–c). The IFG neurons are activated as if the two intervals were of equal duration (Fig. 6II–c), resulting in the time-shrinking illusion. Observations in the IFG indicate the importance of the approximate 50-ms time window after M_3_. Thus, the equality/inequality of two adjacent intervals would be determined by categorical judgment in the right frontal-parietal network (Fig. 6II, middle), and the behavioural results for ATA are derived from activity in this network (Fig. 6II–e). Although we primarily address the encoding, comparison, and judgment processes in terms of temporal judgment tasks, some other functions, especially working memory, are necessary for maintaining and manipulating information online in the absence of incoming sensory stimulation (Fig. 6II, middle). Our findings suggest that activation patterns in two brain areas (TPJ and IFG) are responsible for creating the illusion. To support this claim, we further analysed the data for the T_1<2_ condition (when the ATA effect occurs) by analysing the Equal (illusion) and Unequal (non-illusion) trials for each participant separately. We found a difference between Equal and Unequal activation (Fig. S2 and Table S1). Although further investigation is necessary because of small numbers of trials and participants, the observations from the response-based analyses implicate that activation during illusion trials might differ from non-illusion trials even in physically identical stimulus patterns. Thus, the unique characteristics of the illusion allow us to uncover unprecedented spatiotemporal brain dynamics of the sub-second cognitive temporal processing mechanism. One may argue that temporal information is represented in the SMA^17^, therefore, we need to investigate the involvement of other brain areas, including the SMA, in our task in the future.

## Methods

### Auditory stimuli and procedure

Stimuli were pure 1000-Hz tone bursts lasting 20 ms with rise and fall times of 3 ms, a sampling frequency of 44.1 kHz, and presented using STIM2 software (Neuroscan Co. Ltd., Charlotte, NC, USA). They were amplified and presented diotically to each participant via a pair of inserted earphones (ER-3A, Etymotic Research Inc., Elk Grove Village, IL, USA). We measured the level of a continuous tone using the same amplitude by a sound-level meter with a 1/2-inch condenser microphone (Nos 3431 and 1692; HIOKI, Nagano, Japan) and confirmed that all stimuli were presented at 82 dB SPL.

There were two experimental conditions: judgment and no-judgment. In the former condition, participants judged whether the two durations were equal or unequal by pressing one of two buttons after listening to the M_3_. Participants were instructed to judge only the equivalence of the two intervals. They were not asked to notice the absolute durations. In the no-judgment (control) condition, participants listened to stimuli, and after listening to the M_3_ stimulus, pressed one of the two buttons without making judgments. For both the judgment and no-judgment conditions, the task was divided into seven blocks of 67 trials. Each block contained 18 pseudorandom presentations of the standard stimuli (three patterns × 18 times) and 13 dummy presentations. Dummy presentations were employed to prevent participants from memorising the three target stimulus patterns. The T_1_ and T_2_ intervals for the dummy presentations were selected randomly from a range of 40 to 360 ms, in 40-ms steps. Inter-trial intervals were randomly varied from 3 to 4 s. Allocation of buttons for equal/unequal responses and condition order (judgment/no-judgment) were counterbalanced across participants. Each participant took part in the experiment on two separate days to perform two types of conditions. Participants were instructed to stay alert and to keep their eyes open throughout the experimental blocks.

### Data acquisition

MEG signals were recorded using a 306-channel Neuromag Vectorview MEG system (Elekta, Helsinki, Finland). Anatomical images were obtained using a 3.0 T MRI scanner (Achieve, Philips N.V., Eindhoven, the Netherlands) for analysis and interpretation of MEG data (TE, 60 ms; TR, 100 ms; voxel size, 1.5 × 1.5 × 1.5 mm^3^). Magnetic fields were recorded continuously at a sampling rate of 1000 Hz with a bandpass filter of 0.1–330 Hz. Before each recording, four head-position indicator (HPI) coils attached to the scalp and a 3D digitizer (FastTrack, Polhemus, VT, USA) were used to measure anatomical landmarks (nasion, and left and right auricular points) of the head, the HPI positions, and approximately 200 head-surface points^34^. During the recording, participants were positioned in a quiet magnetically shielded room with their head inside the helmet-shaped sensor array. During each run, continuous HPI (cHPI) measurement was performed using the four HPI coils.

### Signal processing and source reconstruction

MEG signals were obtained from approximately 126 responses for each stimulus pattern (18 trials/block × 7 blocks) in both judgment and no-judgment conditions. All data sets were filtered using Maxfilter to eliminate noise outside of the brain and head-movement correction was applied using the information obtained during cHPI measurement^35^. The cortical surface of each participant was reconstructed using FreeSurfer software^36^. A reconstructed MRI contour was co-registered with the MEG head coordinate system using head-shape points. Signals were bandpass-filtered in the 1–30 Hz range before averaging. Independent component analysis was used to remove human artifacts such as blinks, eye movement, and cardiac signals using a hybrid approach of source localisation and decomposed time-signal analysis^37^. Averaging was performed according to the trigger onset of the first sound. To eliminate outstanding artifacts, trials were excluded during the averaging process if they exceeded a rejection threshold of 5000 fT/cm (8000 fT) for gradiometer (magnetometer) channels. Source localisation was performed for the averaged data using noise-normalised minimum norm estimation (MNE), executed with dynamic statistical parametric mapping (*dSPM*). Specific details of the MNE and *dSPM* algorithms have been reported elsewhere^38, 39^, but briefly: The lead field that models the signal pattern generated by a unit dipole at each location on the cortical surface was calculated using a boundary element method. The general MNE solution is given by:

1 $$W=R{L}^{T}{(LR{L}^{T}+{\lambda }^{2}C)}^{-1}$$

where *R* is a source covariance matrix, *L* is a lead field matrix, *C* is a noise (sensor) covariance matrix, *λ* ^2^ is a regularisation parameter, and ^*T*^ denotes the transpose operator. The noise normalised Z-value (*dSPM*) with covariance matrix *C* is given by:

2 $$dSPM=WM/\sqrt{{(WC{W}^{T})}_{dd}}$$

where *M* is a filtered MEG observation matrix, and ()_dd_ indicates diagonal operation. In this study, the activation at each cortical location was estimated using the normalised *dSPM* values such that the unit of activation was expressed in arbitrary units (a.u.). A covariance matrix *C* was created from the −100 to 0 ms pre-stimulus period (this time range was also used for the baseline correction of source waveform signals (i.e. regional activities)). To compensate for individual differences, we used a standardised brain (MNI-305, fsaverage; Montreal Neurological Institute^40^).

### Marking of inspected ROIs

**Figure 7:**
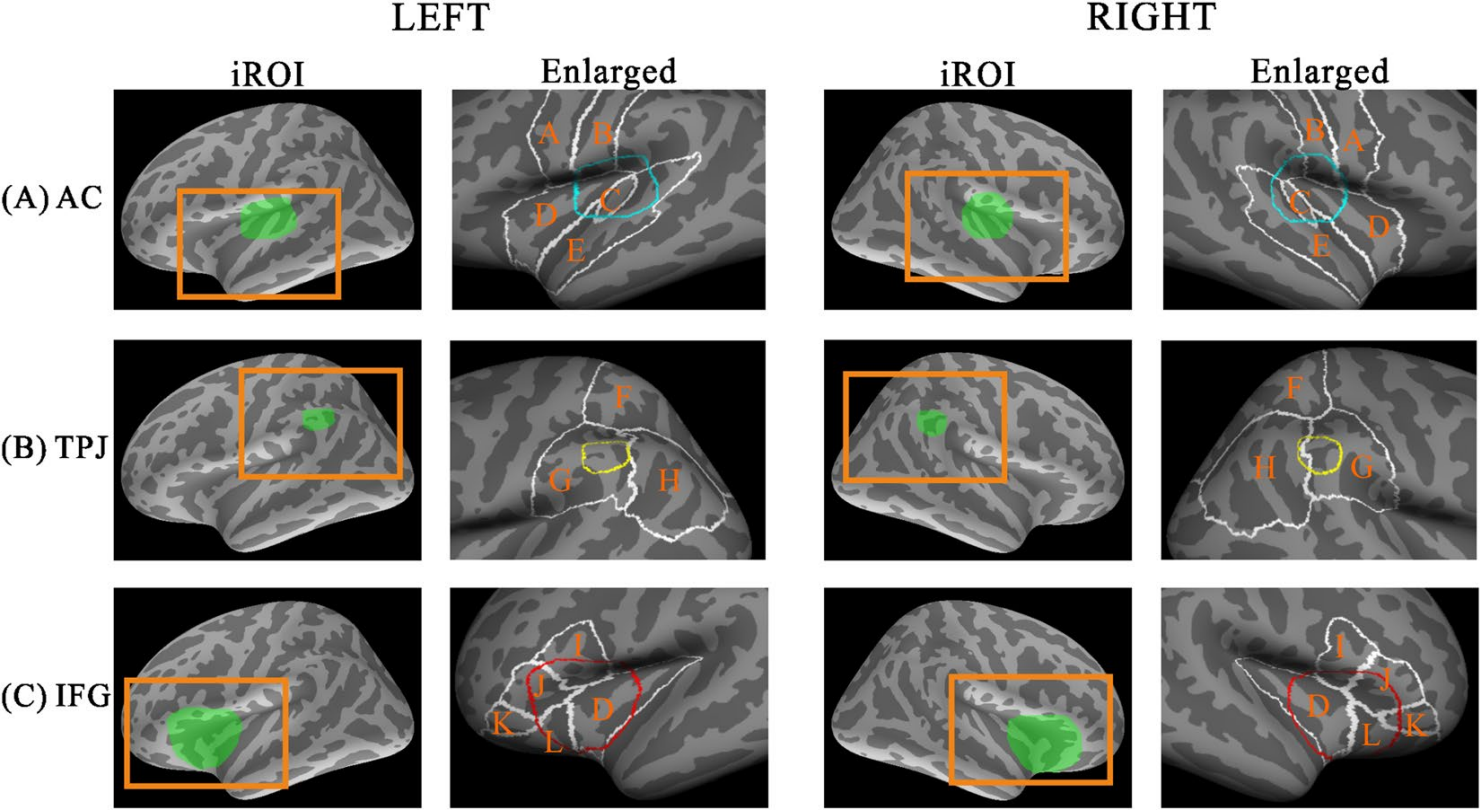
The marked iROIs and adjacent anatomical structures in the left and the right hemispheres for (**A**) the AC, (**B**) the TPJ, and (**C**) IFG. Orange squares represent the adjacent anatomical structures of each iROI (left columns). Enlarged iROIs (right columns) are outlined with cyan for AC, yellow for TPJ, and red for IFG in reference to the white outlined anatomical regions provided by FreeSurfer software (A: precentral, B: postcentral, C: transverse temporal, D: insula, E: superior temporal, F: superior parietal, G: inferior parietal, H: supramarginal, I: pars opercularis, J: pars triangularis, K: pars orbitalis, L: lateral orbitofrontal). The enlarged iROIs (right) with adjacent anatomical structures were subsequently used as activation maps in Fig. 4. iROI; inspected region of interest, AC; auditory cortex, TPJ; temporoparietal junction, IFG; inferior frontal gyrus. The marked iROIs (left) and their enlarged images (right) in both hemispheres.

We first monitored activity in the frontal and parietal regions, as well as in the AC. Estimation was performed according to the methods used in our previous works^41, 42^. To avoid double dipping^43^, we marked iROIs on the AC, IFG, and TPJ of both hemispheres by referencing previous studies and using the anatomical information provided by FreeSurfer software (l(r)h.aparc.annot based on Desikan-Killiany Atlas^44^). For the AC, we set an iROI that covered the transverse temporal gyrus and its immediate vicinity (i.e., the auditory cortex^45^). For the TPJ, the iROI was set between the supramarginal gyrus and inferior parietal lobule^16, 46, 47^. For the IFG, we marked an iROI that covered the inferior frontal gyrus and the superior and anterior insula^13, 48^. Each iROI was first marked in the standard brain and then projected back onto each participant’s cortex. Figure 7 shows the visualisation of marked iROIs used in this study. Anatomical interpretations of the defined target areas in each of three regions both in left and right hemisphere are as follows: the iROI for the AC covered the transverse temporal, supratemporal, and part of the insula (Fig. 7A), the iROI for the TPJ was the superior part of the inferior parietal area (Fig. 7B), and the iROI for the IFG included the pars opercularis, pars triangularis, lateral orbitfrontal cortex, and the insula (Fig. 7C).

### Group analyses

All comparisons of spatial activation maps under certain conditions were created by averaging the *dSPM* results from the 17 participants for the three stimulus patterns after transforming into the standard brain with standardisation. To determine laterality, standardisation was performed by dividing individual data by the maximum peaks from both left and right iROIs, maintaining the left and right activation ratio in each individual participant. Similarly, judgment determination was calculated with standardisation that maintained the judgment and no-judgment activation ratio in each individual. The regional activities in three iROIs for the left and right hemispheres were then compared. To check for hemispheric predominance during temporal processing, the *LI* was calculated at each time point using regional activity from the right and left hemispheres in each iROI:

3 $$L{I}_{iROI}(t)=\frac{dSP{M}_{iROI\_R}(t)-dSP{M}_{iROI\_L}(t)}{dSP{M}_{iROI\_R}(t)+dSP{M}_{iROI\_L}(t)}\,$$

where subscripts *iROI_R* and *iROI_L* represent standardised regional activity (*dSPM*) in the left and right hemisphere in target iROIs, respectively. Subscript *iROI* is either AC, TPJ, or IFG and *t* denotes the instantaneous sampling time point. Similar to the *LI*, a *JI* was calculated at each time point;

4 $$J{I}_{iROI}(t)=\frac{dSP{M}_{iROI\_JT}(t)-dSP{M}_{iROI\_NJ}(t)}{dSP{M}_{iROI\_JT}(t)+dSP{M}_{iROI\_NJ}(t)}\,$$

where subscripts *iROI_JT* and *iROI_NJ* represent standardised regional activity in judgment and no-judgment conditions for the target iROIs. We calculated *JI* in this manner for an evaluation comparable to *LI*. For practical computation of *LI* and *JI*, we used the mean signal across all participants for the regional activity. We assessed these values by calculating the lower limits of their confidence intervals (*CI*s) across the individual participants to find in which iROI, with which stimulus, and at what time points the hemispheric (*LI*) or conditional (*JI*) differences were greater than zero, and reported each index as “*CIs* of *LI”* or “*CIs* of *JI”* (Figs. 2 and 3). For anatomical interpretation of the activity in each iROI, the centre locations for M_3_ responses in the IFG were estimated for the three stimulus patterns in each participant. The activation maps in individual brains were transformed into the standard brain, and centres were estimated for each condition using weighted averaging^34, 41, 42^.

### Statistical analyses

A repeated-measures ANOVA was performed using SPSS Statistics v21 (IBM Inc., Armonk, NY) to assess the behavioural results as well as the amplitudes and latencies of regional activity patterns for the IFG and TPJ in the judgment condition. To analyse the behavioural data in the judgment condition, we conducted a one-way repeated ANOVA using the three standard stimulus patterns as independent variables and the response ratio as the dependent variable after an inverse sine-transformation. Dunnett’s post-hoc *t*-test was performed to check whether the equal response ratios obtained from T_1<2_ and T_1>2_ differed from that for T_1__=__2_. Then, we assessed whether or not hemispheric predominance or conditional differences were present. We conducted the five-way (hemisphere × iROIs × condition × stimulus × time) ANOVA, using the time course of *dSPM* values as outcome measures. For regional activity in the TPJ, a two-way ANOVA was performed on the integrated amplitudes between 80–150 ms after M_2_ for each stimulus pattern in the judgment and no-judgment conditions. For regional activity in the IFG, we assessed peak amplitude and latency corresponding to the M_3_ presentation. An ANOVA was also performed targeting on the integrated amplitude during a 50-ms time window from M_3_ onset, with stimulus pattern as the factor. The Greenhouse–Geisser correction was applied when the assumption of sphericity was violated in the dependent measures. Post-hoc Bonferroni corrections for multiple comparisons were applied when required. The *η* ^2^ *p* (partial eta-squares) were calculated to quantitatively compare effect sizes. Based on our *a priori* hypothesis described in the introduction, we determined three target time windows after applying the following process to investigate the neuromagnetic brain activity related to ATA: For TPJ and IFG, we first set the approximate window with reference to the literature^10, 11, 49^. Within the set window, we marked the peak latencies for each individual and narrowed the window size according to the minimum and maximum values of the selected peaks. Then we applied further statistics while shifting the window parameters. Finally, along with the statistical results, the window size was determined with rounding to the nearest 10-ms.

### Data Availability

All data generated or analysed during this study are included in this published article (and its Supplementary Information files).

## Electronic supplementary material

Supplementary Information

Supplementary Audio1

Supplementary Audio2

Supplementary Audio3

Supplementary Audio4

Supplementary Audio5

Supplementary Audio6
