# Supplementary material for: Spatiotemporal brain dynamics of auditory temporal assimilation
Source: Sci Rep. 2017 Sep 12;7:11400. doi: 10.1038/s41598-017-11631-0 (PMC5595862; doi:10.1038/s41598-017-11631-0)
Supplement: Supplementary file 1 — Supplementary Information [file 41598_2017_11631_MOESM1_ESM.pdf]

Supplementary Information

Spatiotemporal brain dynamics of auditory temporal assimilation

Naruhito Hironaga[1,†], Takako Mitsudo[1,†,*], Mariko Hayamizu[1,2], Yoshitaka Nakajima[3], Hiroshige Takeichi[4], Shozo Tobimatsu[1]

[1]Department of Clinical Neurophysiology, Neurological Institute Faculty of Medicine, Graduate School of Medical Sciences, Kyushu University, 3-1-1 Maidashi, Higashi-ku, Fukuoka 812-8582, Japan. [2]Department of Anesthesiology and Critical Care Medicine, Graduate School of Medical Sciences, Kyushu University, 3-1-1 Maidashi, Higashi-ku, Fukuoka 812-8582, Japan. [3]Department of Human Science/Research Center for Applied Perceptual Science, Faculty of Design, Kyushu University, 4-9-1 Shiobaru, Minami-ku, Fukuoka 815-8540, Japan. [4]Computational Engineering Applications Unit, Advanced Center for Computing and Communication (ACCC), RIKEN, 2-1 Hirosawa, Wako, Saitama 351-0198, Japan.

†Equal contribution

Table of contents:

Supplementary Figure S1

Supplementary Audio 1-6

Supplementary Figure S2

Supplementary Table S1

Supplementary Table S2 ANOVA Table for *dSPM* data

Supplementary Table S3 Results of Multiple Comparisons

Supplementary Table S4 ANOVA Table for TPJ after M_2 response and IFG after M_3 response

Supplementary Table S5 Latencies and amplitudes of M100 after M3 in IFG

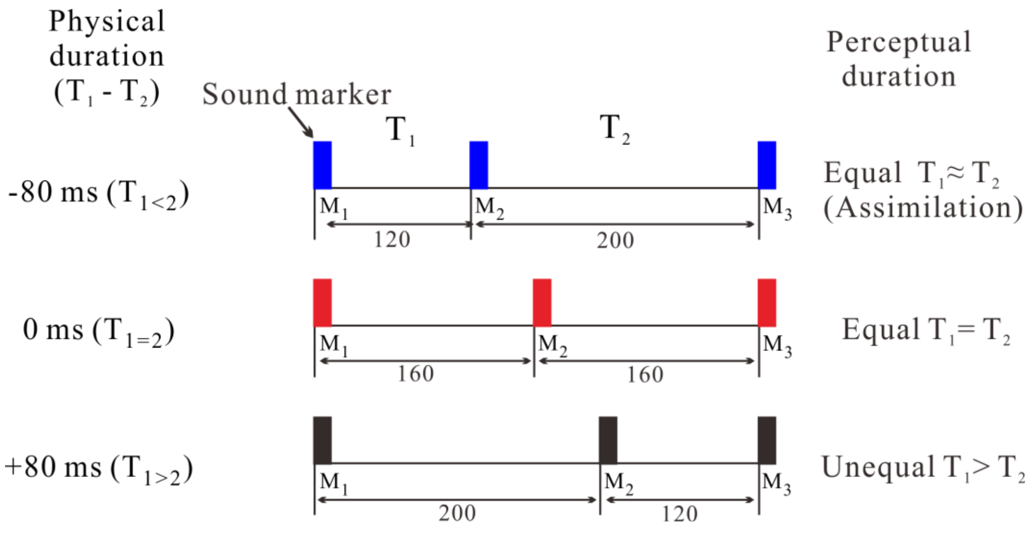

Figure S1. Consider the case in which three successive tone bursts (20 ms) are used to create two adjacent empty (inter-onset) time intervals with durations of 120 ms (T1) and 200 ms (T2) (Supplementary Audio S1). Individuals often perceive the two intervals as equal, despite their substantial difference. This perceptual phenomenon seems to be caused by the auditory temporal assimilation (ATA) process. The magnitude of ATA is markedly greater when the first interval is shorter than the second (Supplementary Audio 1), but not vice versa (Supplementary Audio 3). In perceiving the equality/inequality of two neighbouring time intervals, we should encode the sound markers and compare its duration to the second marker to monitor the passage of time (encoding process). Moreover, we compare the lengths of the two intervals and decide whether they are equal after the third marker (judgment process). ATA enables us to explore the neural processing of duration discrimination in millisecond ranges, which involves cognitive states such as encoding and judgment.

Supplementary Audio

Audio patterns that do or do not cause auditory temporal assimilation (ATA). Readers should listen to Supplementary Audio to appreciate the phenomenon and the behavioural and neurophysiological results. Supplementary Audio 1–3 are for demonstration purposes and 4–6 are the stimuli used in the Experiment. Confirm for yourself that the temporal relationship is only reversed in time between Supplementary Audio 1 and 3, yet 1 and 3 produce different perceptual effects, and that the same T_2 interval of 200 ms apparently sounds different between Supplementary Audio 4 and 6.

Supple-mentary Audio	Physical T_1 (ms)	Physical T_2 (ms)	Total T_1 +T_2 (ms)	Dominant Subjective T_2 (ms)	Auditory Temporal Assimila-tion	Experi-mental Stimulus
1	120	200	320	<200 (time-shrinking¶)	Yes	Demo
2	160	160	320	160	No §	Demo
3	200	120	320	120	No	Demo
4	120	200	320	<200 (time-shrinking¶)	Yes	Stimulus
5	200	200	400	200	No §	Stimulus
6	280	200	480	200	No	Stimulus

¶ see Nakajima et al., 2004[1] for details of time-shrinking.

§ see Miyauchi and Nakajima, 2005[2], 2007[3] for detailed phenomenology in these cases.

Please note that ATA occurs not only in auditory but also in visual[4, 5] and tactile[6] modalities. Therefore, ATA is generally involved in interval judgments for similarly-organised stimuli and unlikely to be related to sensory temporal integration.

References

1 Nakajima, Y. *et al.* Time-shrinking: the process of unilateral temporal assimilation. *Perception* **33**, 1061-1079 (2004).

2 Miyauchi, R. & Nakajima, T. Bilateral assimilation of two neighboring empty time intervals. *Music Perception* **22**, 411-424 (2005).

3 Miyauchi, R. & Nakajima, Y. The category of 1:1 ratio caused by assimilation of two neighboring empty time intervals. *Hum Mov Sci* **26**, 717-727 (2007).

4 Arao, H., Suetomi, D. & Nakajima, Y. Does time-shrinking take place in visual temporal patterns? *Perception* **29**, 819-830 (2000).

5 Nagaike, A. *et al.* 'Time-shrinking perception' in the visual system: a psychophysical and high-density ERP study. *Exp Brain Res* **234**, 3279-3290 (2016).

6 Hasuo, E., Kuroda, T. & Grondin, S. About the time-shrinking illusion in the tactile modality. *Acta Psychol (Amst)* **147**, 122-126 (2014).

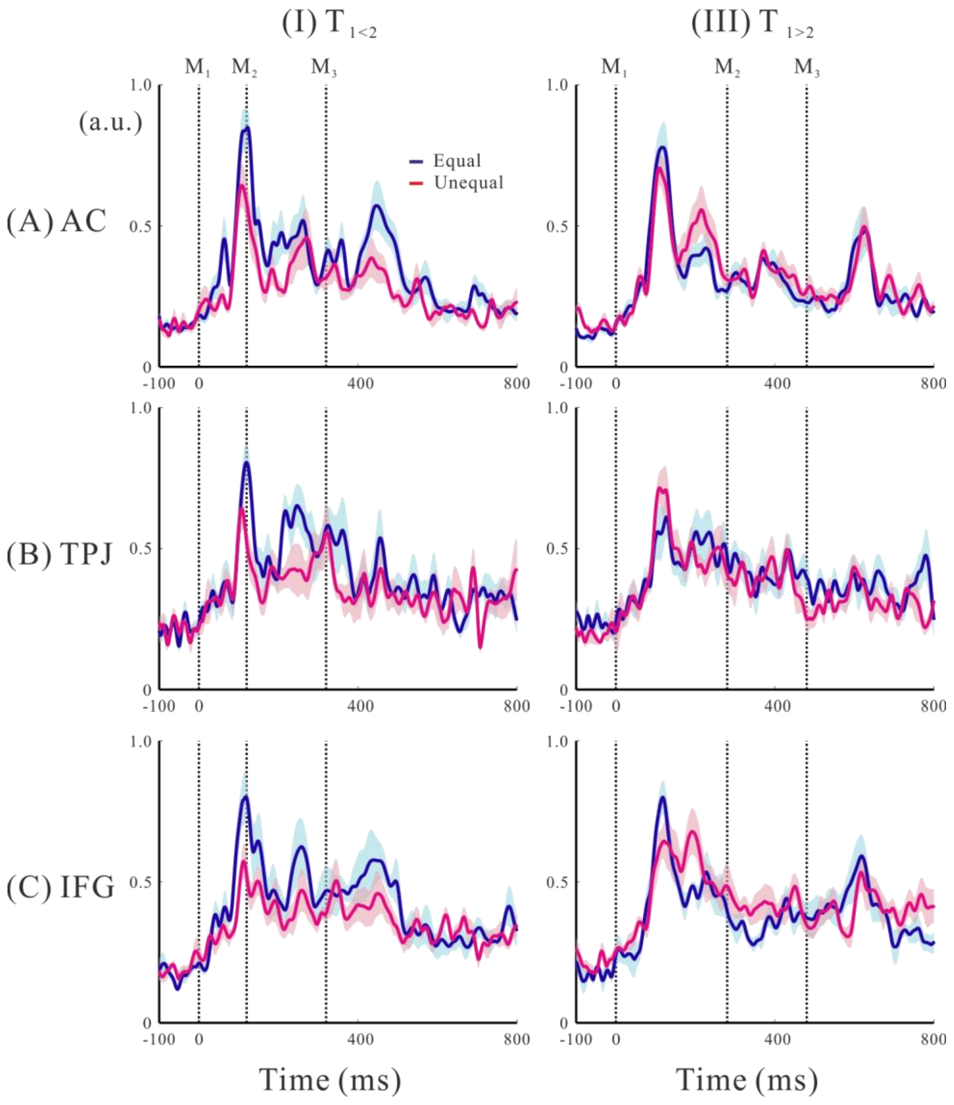

Figure S2. Results of response-based analysis of regional activities in the AC (A), TPJ (B), and IFG (C) for the two different stimulus patterns ($T_{1<2}$ (I) and $T_{1>2}$ (II)). The timing of sound markers is plotted as vertical dotted lines and labelled as M_1 (M; marker), M_2, and M_3. The blue lines represent the time-series data for the equal responses (n = 6 for $T_{1<2}$ (I) and n = 8 for $T_{1>2}$ (III)) regional activity, and the red lines denote those for the unequal responses. Colour-matched transparent areas represent the SEs of the averaged activations.

Table S1. 'Equal' response rate of each participant used in response-based analysis

$T_{1<2}$		$T_{1>2}$	
P3	79.51	P3	55.65
P4	62.83	P5	31.75
P5	82.40	P6	12.90
P10	66.94	P7	67.20
P12	75.40	P8	64.52
P16	82.40	P10	78.05
		P11	66.40
		P16	26.02

In the judgment condition, participants were required to judge whether the T_1 and T_2 durations were equal by pressing one of two buttons. We analysed these responses based on the data sets of the judgment condition in the right hemisphere. We split the judgment trials of $T_{1<2}$ and $T_{1>2}$ based on participants' equal (EQ) and unequal (UE) responses. The data analysis was based on the same procedure used for the left hemisphere and right hemisphere comparison (Fig. 2) and the judgment and no-judgment comparison (Fig. 3). First, we identified the analysable dataset, because some participants' data contained extremely small numbers of equal or unequal responses and were statistically not feasible to analyse. We then set the threshold to be at least 16 trials, because the MNE suit software requires this number to create a stable sensor covariance matrix.

Table S1 summarises the EQ and UE response rates ($T_{1<2}$: n = 6, $T_{1>2}$: n = 8). There were marginally significant differences between EQ/UE responses in $T_{1<2}$ ($F_{1,5} = 5.71$, $p < 0.06$) but not in $T_{1>2}$ ($F_{1,7} = 1.74$, $p = 0.23$). This suggests that the equal judgments in $T_{1<2}$ and $T_{1>2}$ are derived from a different cognitive mechanisms. Note that there were two types of participants who were excluded from this analysis: Data from participants who made responses interpreted as occurrence of the illusion *and* those who made correct, that is, non-illusory responses in

more than 82% of the trials. The behavioural activity associated with the EQ/UE judgment differed between the $T_{1<2}$ and $T_{1>2}$ conditions. This implies that different cognitive processing occurred in the two conditions. In general, sufficient numbers of trials and participants are necessary to report robust evidence. Thus, other approaches are required to confirm that these differential behaviours are indeed associated with the illusion.

Table S2. ANOVA results for *dSPM* data

Factor	SumSq.	d.f.	d.f. Error	MeanSq.	F	$\eta^2 p$	p Value
Hemisphere **	39.66	1	16	39.66	10.02	0.39	0.0060
iROI **	358.57	2	32	179.28	68.32	0.81	0.0000
Stimulus **	59.12	2	32	29.56	37.20	0.70	0.0000
Condition **	15.64	1	16	15.64	9.53	0.37	0.0071
Time **	350.74	4	64	87.69	24.10	0.60	0.0000
Hemisphere × iROI	2.41	2	32	1.21	0.76	0.05	0.4389
Hemisphere × Stimulus	1.75	2	32	0.87	2.14	0.12	0.1540
iROI × Stimulus *	2.18	4	64	0.55	3.33	0.17	0.0203
Hemisphere × Condition	0.03	1	16	0.03	0.05	0.00	0.8215
iROI × Condition	0.29	2	32	0.14	0.25	0.02	0.7343
Stimulus × Condition	0.68	2	32	0.34	0.77	0.05	0.4691
Hemisphere × Time	5.11	4	64	1.28	1.08	0.06	0.3345
iROI × Time **	58.63	8	128	7.33	17.94	0.53	0.0000
Stimulus × Time **	171.25	8	128	21.41	24.13	0.60	0.0000
Condition × Time	0.53	4	64	0.13	0.43	0.03	0.7449
Hemisphere × iROI × Stimulus	0.66	4	64	0.17	1.13	0.07	0.3476
Hemisphere × iROI × Condition	1.27	2	32	0.64	1.36	0.08	0.2710
Hemisphere × Stimulus × Condition	0.16	2	32	0.08	0.52	0.03	0.5633
iROI × Stimulus × Condition	0.77	4	64	0.19	1.03	0.06	0.3823
Hemisphere × iROI × Time	1.27	8	128	0.16	0.73	0.04	0.5533
Hemisphere × Stimulus × Time	3.30	8	128	0.41	1.22	0.07	0.3121
iROI × Stimulus × Time **	16.68	16	256	1.04	6.67	0.29	0.0000
Hemisphere × Condition × Time	1.07	4	64	0.27	1.92	0.11	0.1477
iROI × Condition × Time	1.08	8	128	0.14	1.06	0.06	0.3783
Stimulus × Condition × Time	1.66	8	128	0.21	1.04	0.06	0.3978
Hemisphere × iROI × Stimulus × Condition	0.08	4	64	0.02	0.22	0.01	0.8952
Hemisphere × iROI × Stimulus × Time	3.35	16	256	0.21	1.71	0.10	0.1125
Hemisphere ×iROI × Condition × Time	0.68	8	128	0.09	0.75	0.05	0.5750
Hemisphere × Stimulus × Condition × Time	0.41	8	128	0.05	0.48	0.03	0.7347
iROI × Stimulus × Condition × Time	1.53	16	256	0.10	1.13	0.07	0.3526
Hemisphere × iROI × Stimulus × Condition × Time	1.00	16	256	0.06	0.68	0.04	0.6729

The results of the five-way analysis of variance (ANOVA) for *dSPM* values are shown. The major factors were Hemisphere (2 levels: Left and Right), iROI (3 levels: Auditory Cortex, Temporo-Parietal Junction, and Inferior Frontal Gyrus), Stimulus Pattern (3 levels: $T_{1<2}$, $T_{1=2}$, $T_{1>2}$), Condition (2 levels: Judgment and No Judgment), and Time (5 levels: the first interval, the first 100 ms of the second interval, the second 100 ms of the second interval, the first 100 ms after the stimulus period, and the last 800 ms after stimulus onset). All interactions were included in this analysis. As a result, main effects for all the factors were significant at the $p < 0.01$ level (**). Additionally, the iROI × Stimulus × Time, iROI × Time, and Stimulus × Time

interactions were significant at the $p < 0.01$ level. The iROI × Stimulus interaction was significant at the $p < 0.05$ level (*).

Table S3. Results of multiple comparisons

a. Auditory Cortex (AC)

Term 1	Term 2	Difference	StdErr	pValue	Lower	Upper
AC $T_{1<2}$ T1	AC $T_{1<2}$ PostStimulus Late	1.15	0.19	0.0066	0.23	2.07
AC $T_{1<2}$ T1	AC $T_{1=2}$ PostStimulus Early	1.27	0.23	0.0124	0.19	2.35
AC $T_{1<2}$ T1	AC $T_{1>2}$ PostStimulus Early	1.30	0.21	0.0038	0.31	2.29
AC $T_{1<2}$ T2 Early	AC $T_{1<2}$ PostStimulus Late	2.09	0.28	0.0005	0.77	3.41
AC $T_{1<2}$ T2 Early	AC $T_{1<2}$ PostStimulus Early	1.66	0.32	0.0247	0.13	3.18
AC $T_{1<2}$ T2 Early	AC $T_{1=2}$ PostStimulus Late	1.92	0.28	0.0014	0.59	3.24
AC $T_{1<2}$ T2 Early	AC $T_{1=2}$ T2 Early	1.23	0.23	0.0159	0.15	2.30
AC $T_{1<2}$ T2 Early	AC $T_{1=2}$ PostStimulus Early	2.21	0.29	0.0004	0.83	3.59
AC $T_{1<2}$ T2 Early	AC $T_{1>2}$ PostStimulus Late	1.83	0.30	0.0050	0.40	3.26
AC $T_{1<2}$ T2 Early	AC $T_{1>2}$ T1	0.69	0.13	0.0216	0.07	1.32
AC $T_{1<2}$ T2 Early	AC $T_{1>2}$ T2 Early	1.76	0.27	0.0025	0.47	3.04
AC $T_{1<2}$ T2 Early	AC $T_{1>2}$ T2 Late	1.67	0.33	0.0307	0.10	3.25
AC $T_{1<2}$ T2 Early	AC $T_{1>2}$ PostStimulus Early	2.24	0.29	0.0004	0.86	3.62
AC $T_{1<2}$ T2 Late	AC $T_{1<2}$ PostStimulus Late	0.69	0.13	0.0244	0.06	1.33
AC $T_{1<2}$ T2 Late	AC $T_{1=2}$ PostStimulus Early	0.81	0.13	0.0054	0.18	1.45
AC $T_{1<2}$ T2 Late	AC $T_{1>2}$ PostStimulus Early	0.84	0.16	0.0256	0.06	1.62
AC $T_{1<2}$ PostStimulus Early	AC $T_{1<2}$ PostStimulus Late	0.43	0.08	0.0201	0.04	0.82
AC $T_{1<2}$ PostStimulus Early	AC $T_{1=2}$ PostStimulus Early	0.55	0.05	0.0000	0.29	0.81
AC $T_{1<2}$ PostStimulus Early	AC $T_{1>2}$ PostStimulus Early	0.58	0.09	0.0046	0.13	1.03
AC $T_{1=2}$ T1	AC $T_{1<2}$ PostStimulus Late	1.62	0.21	0.0004	0.61	2.64
AC $T_{1=2}$ T1	AC $T_{1=2}$ PostStimulus Late	1.45	0.22	0.0027	0.39	2.51
AC $T_{1=2}$ T1	AC $T_{1=2}$ PostStimulus Early	1.74	0.24	0.0007	0.61	2.87
AC $T_{1=2}$ T1	AC $T_{1>2}$ PostStimulus Late	1.36	0.24	0.0088	0.24	2.48
AC $T_{1=2}$ T1	AC $T_{1>2}$ T2 Early	1.29	0.22	0.0062	0.26	2.31
AC $T_{1=2}$ T1	AC $T_{1>2}$ PostStimulus Early	1.77	0.23	0.0003	0.69	2.85
AC $T_{1=2}$ T2 Early	AC $T_{1<2}$ PostStimulus Late	0.86	0.17	0.0236	0.07	1.65
AC $T_{1=2}$ T2 Early	AC $T_{1=2}$ PostStimulus Early	0.98	0.14	0.0014	0.30	1.66
AC $T_{1=2}$ T2 Early	AC $T_{1>2}$ PostStimulus Early	1.01	0.15	0.0015	0.31	1.72
AC $T_{1=2}$ T2 Late	AC $T_{1=2}$ PostStimulus Early	0.64	0.13	0.0441	0.01	1.27
AC $T_{1>2}$ PostStimulus Late	AC $T_{1>2}$ PostStimulus Early	0.41	0.08	0.0199	0.04	0.77
AC $T_{1>2}$ T1	AC $T_{1<2}$ PostStimulus Late	1.40	0.18	0.0004	0.53	2.27
AC $T_{1>2}$ T1	AC $T_{1=2}$ PostStimulus Late	1.23	0.20	0.0055	0.26	2.19
AC $T_{1>2}$ T1	AC $T_{1=2}$ PostStimulus Early	1.52	0.20	0.0005	0.56	2.48
AC $T_{1>2}$ T1	AC $T_{1>2}$ PostStimulus Late	1.14	0.21	0.0151	0.15	2.13
AC $T_{1>2}$ T1	AC $T_{1>2}$ T2 Early	1.07	0.18	0.0059	0.22	1.91
AC $T_{1>2}$ T1	AC $T_{1>2}$ PostStimulus Early	1.55	0.19	0.0002	0.64	2.46
AC $T_{1>2}$ T2 Early	AC $T_{1>2}$ PostStimulus Early	0.48	0.09	0.0181	0.05	0.91

b. Temporo-Parietal Junction (TPJ)

Term 1	Term 2	Difference	StdErr	pValue	Lower	Upper
TPJ $T_{1<2}$ T2 Early	TPJ $T_{1<2}$ PostStimulus Late	1.06	0.15	0.0008	0.36	1.77
TPJ $T_{1<2}$ T2 Early	TPJ $T_{1<2}$ T1	0.61	0.10	0.0062	0.12	1.09
TPJ $T_{1<2}$ T2 Early	TPJ $T_{1=2}$ PostStimulus Late	1.11	0.15	0.0005	0.40	1.81
TPJ $T_{1<2}$ T2 Early	TPJ $T_{1=2}$ T1	0.36	0.06	0.0031	0.09	0.63
TPJ $T_{1<2}$ T2 Early	TPJ $T_{1=2}$ T2 Early	0.67	0.13	0.0190	0.07	1.27

**TPJ $T_{1<2}$ T2 Early**	**TPJ $T_{1=2}$ T2 Late**	**0.93**	**0.19**	**0.0410**	**0.02**	**1.83**
**TPJ $T_{1<2}$ T2 Early**	**TPJ $T_{1=2}$ PostStimulus Early**	**0.99**	**0.16**	**0.0037**	**0.24**	**1.73**
**TPJ $T_{1<2}$ T2 Early**	**TPJ $T_{1>2}$ PostStimulus Late**	**1.05**	**0.15**	**0.0009**	**0.35**	**1.75**
**TPJ $T_{1<2}$ T2 Early**	**TPJ $T_{1>2}$ T1**	**0.49**	**0.07**	**0.0018**	**0.14**	**0.83**
**TPJ $T_{1<2}$ T2 Early**	**TPJ $T_{1>2}$ T2 Early**	**0.88**	**0.15**	**0.0057**	**0.19**	**1.58**
**TPJ $T_{1<2}$ T2 Early**	**TPJ $T_{1>2}$ T2 Late**	**0.95**	**0.15**	**0.0032**	**0.24**	**1.66**
**TPJ $T_{1<2}$ T2 Early**	**TPJ $T_{1>2}$ PostStimulus Early**	**1.11**	**0.17**	**0.0021**	**0.31**	**1.91**
**TPJ $T_{1<2}$ T2 Late**	**TPJ $T_{1<2}$ PostStimulus Late**	**0.50**	**0.08**	**0.0032**	**0.13**	**0.88**
**TPJ $T_{1<2}$ T2 Late**	**TPJ $T_{1=2}$ PostStimulus Late**	**0.55**	**0.10**	**0.0131**	**0.08**	**1.02**
**TPJ $T_{1<2}$ T2 Late**	**TPJ $T_{1=2}$ PostStimulus Early**	**0.42**	**0.08**	**0.0110**	**0.07**	**0.78**
**TPJ $T_{1<2}$ T2 Late**	**TPJ $T_{1>2}$ PostStimulus Late**	**0.49**	**0.09**	**0.0146**	**0.07**	**0.92**
**TPJ $T_{1<2}$ T2 Late**	**TPJ $T_{1>2}$ PostStimulus Early**	**0.55**	**0.11**	**0.0465**	**0.00**	**1.09**
**TPJ $T_{1<2}$ PostStimulus Early**	**TPJ $T_{1<2}$ PostStimulus Late**	**0.39**	**0.06**	**0.0021**	**0.11**	**0.67**
**TPJ $T_{1<2}$ PostStimulus Early**	**TPJ $T_{1=2}$ PostStimulus Late**	**0.44**	**0.07**	**0.0043**	**0.10**	**0.77**
**TPJ $T_{1<2}$ PostStimulus Early**	**TPJ $T_{1=2}$ PostStimulus Early**	**0.32**	**0.05**	**0.0011**	**0.10**	**0.53**
**TPJ $T_{1<2}$ PostStimulus Early**	**TPJ $T_{1>2}$ PostStimulus Late**	**0.38**	**0.06**	**0.0035**	**0.09**	**0.67**
**TPJ $T_{1<2}$ PostStimulus Early**	**TPJ $T_{1>2}$ PostStimulus Early**	**0.44**	**0.08**	**0.0199**	**0.04**	**0.83**
**TPJ $T_{1=2}$ T1**	**TPJ $T_{1<2}$ PostStimulus Late**	**0.70**	**0.14**	**0.0270**	**0.05**	**1.36**
**TPJ $T_{1=2}$ T1**	**TPJ $T_{1=2}$ PostStimulus Late**	**0.75**	**0.14**	**0.0150**	**0.10**	**1.40**
**TPJ $T_{1=2}$ T1**	**TPJ $T_{1>2}$ PostStimulus Late**	**0.69**	**0.14**	**0.0295**	**0.04**	**1.34**
**TPJ $T_{1=2}$ T1**	**TPJ $T_{1>2}$ PostStimulus Early**	**0.75**	**0.15**	**0.0353**	**0.03**	**1.46**
**TPJ $T_{1>2}$ T1**	**TPJ $T_{1<2}$ PostStimulus Late**	**0.58**	**0.11**	**0.0142**	**0.08**	**1.08**
**TPJ $T_{1>2}$ T1**	**TPJ $T_{1<2}$ T2 Early**	**-0.49**	**0.07**	**0.0018**	**-0.83**	**-0.14**
**TPJ $T_{1>2}$ T1**	**TPJ $T_{1=2}$ PostStimulus Late**	**0.62**	**0.10**	**0.0049**	**0.14**	**1.11**
**TPJ $T_{1>2}$ T1**	**TPJ $T_{1>2}$ PostStimulus Late**	**0.57**	**0.10**	**0.0094**	**0.10**	**1.04**
**TPJ $T_{1>2}$ T1**	**TPJ $T_{1>2}$ PostStimulus Early**	**0.62**	**0.12**	**0.0282**	**0.04**	**1.21**

c. Inferior Frontal Gyrus (IFG)

**Term 1**	**Term 2**	**Difference**	**StdErr**	**pValue**	**Lower**	**Upper**
**IFG $T_{1<2}$ T1**	**IFG $T_{1<2}$ PostStimulus Late**	**0.50**	**0.10**	**0.0215**	**0.05**	**0.96**
**IFG $T_{1<2}$ T2 Early**	**IFG $T_{1<2}$ PostStimulus Late**	**1.39**	**0.19**	**0.0005**	**0.51**	**2.28**
**IFG $T_{1<2}$ T2 Early**	**IFG $T_{1<2}$ T1**	**0.89**	**0.18**	**0.0292**	**0.06**	**1.73**
**IFG $T_{1<2}$ T2 Early**	**IFG $T_{1<2}$ PostStimulus Early**	**0.93**	**0.19**	**0.0348**	**0.04**	**1.83**
**IFG $T_{1<2}$ T2 Early**	**IFG $T_{1=2}$ PostStimulus Late**	**1.30**	**0.17**	**0.0003**	**0.52**	**2.08**
**IFG $T_{1<2}$ T2 Early**	**IFG $T_{1=2}$ T1**	**0.50**	**0.10**	**0.0338**	**0.02**	**0.97**
**IFG $T_{1<2}$ T2 Early**	**IFG $T_{1=2}$ T2 Early**	**0.76**	**0.14**	**0.0204**	**0.08**	**1.45**
**IFG $T_{1<2}$ T2 Early**	**IFG $T_{1=2}$ T2 Late**	**1.02**	**0.17**	**0.0045**	**0.23**	**1.80**
**IFG $T_{1<2}$ T2 Early**	**IFG $T_{1=2}$ PostStimulus Early**	**1.36**	**0.16**	**0.0001**	**0.58**	**2.15**
**IFG $T_{1<2}$ T2 Early**	**IFG $T_{1>2}$ PostStimulus Late**	**1.19**	**0.19**	**0.0035**	**0.30**	**2.09**
**IFG $T_{1<2}$ T2 Early**	**IFG $T_{1>2}$ T1**	**0.56**	**0.10**	**0.0102**	**0.09**	**1.02**
**IFG $T_{1<2}$ T2 Early**	**IFG $T_{1>2}$ T2 Early**	**1.19**	**0.18**	**0.0019**	**0.34**	**2.03**
**IFG $T_{1<2}$ T2 Early**	**IFG $T_{1>2}$ PostStimulus Early**	**1.35**	**0.18**	**0.0004**	**0.52**	**2.19**
**IFG $T_{1<2}$ T2 Late**	**IFG $T_{1<2}$ PostStimulus Late**	**0.61**	**0.09**	**0.0012**	**0.19**	**1.03**
**IFG $T_{1<2}$ T2 Late**	**IFG $T_{1=2}$ PostStimulus Late**	**0.52**	**0.09**	**0.0121**	**0.08**	**0.96**
**IFG $T_{1<2}$ T2 Late**	**IFG $T_{1=2}$ PostStimulus Early**	**0.59**	**0.09**	**0.0028**	**0.15**	**1.02**
**IFG $T_{1<2}$ T2 Late**	**IFG $T_{1>2}$ PostStimulus Late**	**0.41**	**0.06**	**0.0030**	**0.11**	**0.72**
**IFG $T_{1<2}$ T2 Late**	**IFG $T_{1>2}$ T2 Early**	**0.41**	**0.08**	**0.0442**	**0.01**	**0.81**
**IFG $T_{1<2}$ T2 Late**	**IFG $T_{1>2}$ PostStimulus Early**	**0.57**	**0.10**	**0.0063**	**0.12**	**1.03**
**IFG $T_{1<2}$ PostStimulus Early**	**IFG $T_{1=2}$ PostStimulus Early**	**0.43**	**0.05**	**0.0002**	**0.18**	**0.68**
**IFG $T_{1=2}$ T1**	**IFG $T_{1<2}$ PostStimulus Late**	**0.90**	**0.14**	**0.0024**	**0.25**	**1.55**
**IFG $T_{1=2}$ T1**	**IFG $T_{1=2}$ PostStimulus Late**	**0.80**	**0.12**	**0.0025**	**0.22**	**1.39**
**IFG $T_{1=2}$ T1**	**IFG $T_{1=2}$ PostStimulus Early**	**0.87**	**0.13**	**0.0018**	**0.25**	**1.48**
**IFG $T_{1=2}$ T1**	**IFG $T_{1>2}$ T2 Early**	**0.69**	**0.13**	**0.0145**	**0.09**	**1.29**

**IFG $T_{1=2}$ T1**	**IFG $T_{1>2}$ PostStimulus Early**	**0.86**	**0.13**	**0.0026**	**0.23**	**1.49**
**IFG $T_{1=2}$ T2 Early**	**IFG $T_{1<2}$ PostStimulus Late**	**0.63**	**0.13**	**0.0338**	**0.03**	**1.24**
**IFG $T_{1=2}$ T2 Early**	**IFG $T_{1=2}$ PostStimulus Late**	**0.54**	**0.11**	**0.0280**	**0.04**	**1.04**
**IFG $T_{1=2}$ T2 Early**	**IFG $T_{1=2}$ PostStimulus Early**	**0.60**	**0.09**	**0.0017**	**0.18**	**1.03**
**IFG $T_{1=2}$ T2 Early**	**IFG $T_{1>2}$ PostStimulus Early**	**0.59**	**0.10**	**0.0056**	**0.13**	**1.06**
**IFG $T_{1=2}$ T2 Late**	**IFG $T_{1=2}$ PostStimulus Early**	**0.35**	**0.06**	**0.0169**	**0.04**	**0.66**
**IFG $T_{1>2}$ T1**	**IFG $T_{1<2}$ PostStimulus Late**	**0.84**	**0.12**	**0.0013**	**0.26**	**1.41**
**IFG $T_{1>2}$ T1**	**IFG $T_{1=2}$ PostStimulus Late**	**0.74**	**0.11**	**0.0014**	**0.23**	**1.26**
**IFG $T_{1>2}$ T1**	**IFG $T_{1=2}$ PostStimulus Early**	**0.81**	**0.11**	**0.0008**	**0.27**	**1.34**
**IFG $T_{1>2}$ T1**	**IFG $T_{1>2}$ PostStimulus Late**	**0.64**	**0.13**	**0.0289**	**0.04**	**1.24**
**IFG $T_{1>2}$ T1**	**IFG $T_{1>2}$ T2 Early**	**0.63**	**0.11**	**0.0078**	**0.12**	**1.14**
**IFG $T_{1>2}$ T1**	**IFG $T_{1>2}$ PostStimulus Early**	**0.80**	**0.11**	**0.0007**	**0.28**	**1.32**

This table lists the significant ($p < 0.05$) pairwise contrasts for the iROI × Stimulus × Time interaction in the five-way ANOVA using the Tukey-Kramer method, which corrects for family-wise multiple comparisons. There were 990 contrasts, among which 440 (44%) were significant. Only contrasts between the same iROI are shown. Note that the Table includes several significant contrasts for early and late T_2 activity in the TPJ, and post-stimulus activity in the IFG, suggesting complex stimulus-dependent spatiotemporal dynamics in brain activation under ATA. *AC*, auditory cortex; *TPJ*, temporo-parietal junction; *IFG*, inferior frontal gyrus; *T_1 and T_2*, the first and the second consecutive intervals marked by three tones; *T_1*, *T_2 Early, T_2 Late, Poststimulus Early, and Poststimulus Late,* the five periods (see legends of Table S2 in Supplementary Information for their definitions); *$T_{1<2}$, $T_{1=2}$, $T_{1>2}$*, the stimulus patterns.

Table S4 ANOVA results for TPJ after M_2 response and IFG after M_3 response.

	Factors	SumSq.	*d.f.*	*d.f.* Err.	Mean Sq.	*F*	$\eta^2 p$	*p* Value
TPJ	Stimulus**	1173.928	1.858	32	631.755	8.298	0.3415	0.0012
	Condition+	311.589	1	16	311.589	4.312	0.2123	0.0543
	Stimulus × Condition	96.974	1.877	32	51.672	1.144	0.0667	0.3312
IFG	Stimulus	117.781	2	32	70.377	4.299	0.2118	0.0222

This table shows the results of the two-way ANOVA on the *dSPM* for the right-TPJ values that are plotted in Figure 5A and the one-way ANOVA for the right-IFG values that are plotted in Figure 5B. For the two-way ANOVA, the major factors were Stimulus Pattern (3 levels: $T_{1<2}$, $T_{1=2}$, $T_{1>2}$) and Condition (2 levels: Judgment and No Judgment). The one-way ANOVA looked at Stimulus Pattern (3 levels: $T_{1<2}$, $T_{1=2}$, $T_{1>2}$). In the right TPJ, we observed a significant main effect of Stimulus ($p < 0.01$, **) and a marginally non-significant main effect of Condition ($p = 0.054$, $^{+}$). Multiple comparisons among the stimulus factors revealed that the integrated amplitude within 70 ms after M_2 was significantly larger for the $T_{1<2}$ pattern than for the $T_{1=2}$ ($p < 0.01$) and $T_{1>2}$ ($p = 0.035$) stimulus patterns. In the right IFG, the main effect of stimulus pattern was significant ($F_{2,32} = 4.29$, $p < 0.03$, $\eta^2 p = 0.21$). The integrated regional activity amplitudes for the $T_{1<2}$ pattern were significantly larger than those for the $T_{1=2}$ ($p = 0.053$) and $T_{1>2}$ ($p = 0.029$) patterns.

Table S5. Latencies and amplitudes of M100 after M_3 in the IFG.

	$T_{1<2}$			$T_{1=2}$			$T_{1>2}$		
	Latency	After adjustment	Amplitude	Latency	After adjustment	Amplitude	Latency	After adjustment	Amplitude
P1	435	115	0.28	527	127	0.30	590	110	0.49
P2	422	102	0.82	513	113	0.53	606	126	0.63
P3	429	109	0.72	531	131	0.72	615	135	1.00
P4	494	174	0.74	553	153	0.83	646	166	0.62
P5	463	143	0.44	559	159	0.90	621	141	0.81
P6	472	152	0.24	587	187	0.48	671	191	0.34
P7	472	152	0.62	549	149	0.68	618	138	0.48
P8	487	167	0.49	566	166	0.48	635	155	0.68
P9	490	170	0.36	528	128	0.48	608	128	0.49
P10	451	131	0.77	531	131	0.78	602	122	0.64
P11	465	145	0.50	548	148	0.39	654	174	0.47
P12	426	106	0.69	554	154	0.48	624	144	0.39
P13	465	145	0.66	528	128	0.67	612	132	0.38
P14	443	123	0.55	527	127	0.67	597	117	0.55
P15	491	171	0.41	541	141	0.44	618	138	0.46
P16	493	173	0.32	565	165	0.61	654	174	0.48
P17	426	106	0.48	501	101	0.53	600	120	0.68
Mean (±SD)	460.2 (26.1)	140.2 (26.1)	0.53 (0.18)	541.6 (21.5)	141.6 (21.5)	0.59 (0.17)	621.8 (22.9)	141.8 (22.9)	0.56 (0.17)

The individual peak amplitudes and latencies within the 101–191-ms time window that are plotted in Figures 5B-(b) and 5B-(c) are shown.
